# Ophiopogonin-B Suppresses Epithelial-mesenchymal Transition in Human Lung Adenocarcinoma Cells via the Linc00668/miR-432-5p/EMT Axis: Erratum

**DOI:** 10.7150/jca.98604

**Published:** 2024-06-01

**Authors:** Cheng Hu, Rilei Jiang, Ziyu Cheng, Yueyang Lu, Ling Gu, Hongxiao Li, Liqiu Li, Qian Gao, Meijuan Chen, Xu Zhang

**Affiliations:** 1 School of Medicine and Life Sciences, Nanjing University of Chinese Medicine, Nanjing, 210023, P.R. China; 2 Jiangsu Collaborative Innovation Center of Traditional Chinese Medicine (TCM) Prevention and Treatment of Tumor, Nanjing University of Chinese Medicine, Nanjing, 210023, P.R. China

We recently have noticed two inadvertent mistakes due to our carelessness and high similar images in the preparation of the figures and would like to correct them.

1. The western blot band of actin in Figure 1A was misplaced. We had replaced the band of actin in Figure 1A.
2. The images in Figure 1C were misused. We had replaced the images in Figure 1C and the corresponding statistical charts (Figure 1D) are also updated and the conclusion does not change.

The correction does not change the overall conclusions of this paper. All of the data have been checked carefully, and no errors exist in the corrected version.

The authors apologize for any inconvenience caused by this incidence.

## Figures and Tables

**Figure 1:**
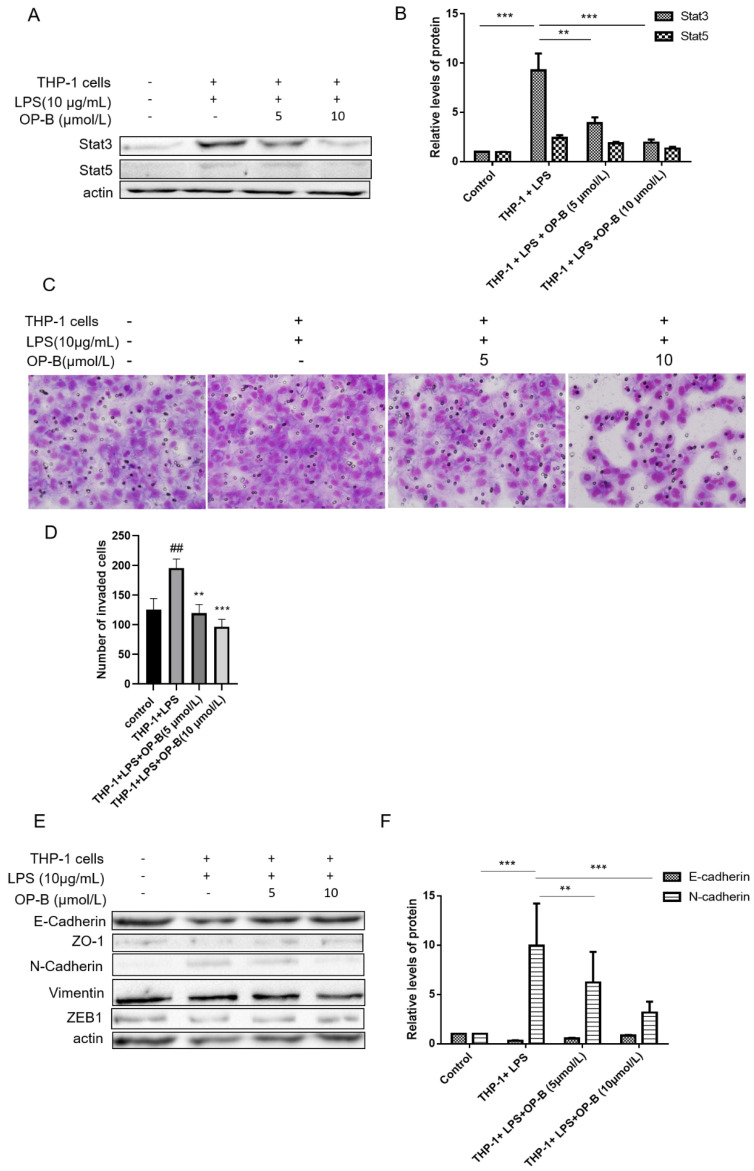
OP-B inhibits the migration of A549 cells co-cultured with LPS-challenged THP-1 cells. (A~B), Levels of Stat3 and Stat5 were detected by western blot assay with specific antibodies, β-actin was used as a control. (C~D), Effects of OP-B on the migration of cells across the Transwell chamber in the co-culture system. Images are representative of three independent experiments. (E~F), Effects of OP-B on the levels of EMT-related markers in co-cultured A549 cells. Levels of E-cadherin, ZO-1, N-cadherin, ZEB1, and Vimentin were detected by western blot assay, β-actin was used as a loading control. Error bars represented mean ± SD from three independent experiments. (^##^), *P*< 0.01; (^**^), *P* < 0.01; (^***^), *P* < 0.001.

